# Spine injections: the rationale for CT guidance

**DOI:** 10.1007/s00256-022-04188-1

**Published:** 2022-09-23

**Authors:** Sanja Bogdanovic, Reto Sutter, Veronika Zubler

**Affiliations:** Department of Radiology, Orthopedic University Hospital Balgrist, Zurich, Forchstrasse 340, 8008 Zurich, Switzerland

**Keywords:** CT-guided spine injections, Epidural injection, Nerve root block, Steroid injections

## Abstract

Back pain is one of the most common medical problems and is associated with high socioeconomic costs. Imaging-guided spinal injections are a minimally invasive method to evaluate where the back pain is originating from, and to treat patients with radicular pain or spinal stenosis with infiltration of corticosteroids. CT-guided spine injections are a safe procedure, characterized by precise needle placement, excellent visualization of the relevant anatomical structures, and low radiation exposure for the patient and the interventional radiologist. In this review article, the variety of applications of CT-guided injections (focused on nerve roots and epidural injections) and the optimal injection procedure as well as risks and side effects are discussed.

## Introduction

Back pain is one of the most common global health problems and among the most frequent causes for disability-adjusted life years (DALYs) from adolescence to adulthood [1]. Back pain is most frequently located in the lower back, followed by the neck, whereas thoracic back pain is less common [2]. There are many causes of back pain, e.g., poor posture, muscle problems, fragility fractures, or nerve root irritation [3], and there are various therapeutical approaches to treat them. While muscle problems are often self-limiting, back pain resulting from nerve root irritation caused by disk herniation or other degenerative changes of the spine creates substantial and persistent morbidity [4, 5]. Patients with acute motor palsy need to undergo surgery, but most patients do not have motor symptoms, and are only seeking medical treatment after experiencing pain for several days or even weeks. In these patients, the pain is often treated non-operatively, e.g., with physical therapy, oral medication, or image-guided spinal injections. In patients with lumbar disk herniations, minimally invasive nerve root injections with corticosteroids are usually very successful and can reduce the pain significantly, with 62.7% of patients reporting being “much better” or “better” 4 weeks after the injection, and an average pain reduction of 3.4 on the numeric rating scale (*p* = 0.0001) [6]. It was further shown that MRI findings correlated with outcomes after transforaminal epidural steroid injections: The most significant pain relief after 4 weeks was reported for patients with disk protrusion plus sequestration, with an average pain reduction of 3.1 on the numeric rating scale (*p* = 0.0001) [7]. Furthermore, successful nerve root injections may be sufficient to reduce a patient’s pain and delay decompressive surgery up to 28 months which leads to less morbidity and mortality and less financial expenditure [8]. Gugliotta et al. showed in a prospective cohort study that patients treated with an open discectomy experienced a faster pain relief than those treated with non-operative methods like physical therapy, oral medication, or CT-guided periradicular infiltrations: After 6 weeks, 48% of patients with surgery reported a ≥ 50% decrease in back pain symptoms, compared to only 17% of patients with non-operative therapy. But the difference between the operative and non-operative group vanished after 3 months. The authors stated, however, that a substantial number of patients were lost to follow-up [9].

In this review article, we focus on CT-guided epidural injections and nerve root injections: Why, when, and how should we perform these spinal interventions under CT guidance. Other spine procedures like injections of facet joints, sacroiliac joints, or isthmic spondylolysis are not discussed.

## CT guidance: rationale and radiation exposure

Several factors may influence the decision which image modality to use for nerve root blocks or epidural injections: the availability of examination slots in the CT unit or fluoroscopy unit, considerations about safety and radiation dose, or preference by the interventionalist or the referring physician [10–15]. Additionally, cost considerations and waiting times often play a role in the choice of modality [16].

Some physicians perform blind injections without any imaging guidance, relying on the palpation of landmarks such as the spinous process and the iliac crest, and on the loss-of-resistance technique when advancing the needle [17, 18]. Image-guided injections, however, are superior in reaching the correct anatomical target and documenting the needle placement and contrast distribution, and they allow the identification of inadvertent punctures and the subsequent correction of the needle position [19–21]. Apart from CT and fluoroscopy, also ultrasound or MRI has been used for needle guidance in spinal injections [22–25], but less frequently.

The positioning of the needle under CT guidance is simple, because the location of the needle tip as well as the anatomic structures can be directly visualized during nerve root injections and epidural injections. A short planning CT allows measuring the distance between the planned insertion point on the skin and the target and choosing the appropriate needle for every patient.

At our institution, the training of new fellows on the CT-guided spine injections by the staff radiologists is straightforward, as evidenced both by our personal experience and by the equal success rates in patient outcome for injections performed by the fellows and the more experienced radiologists [26].

The spine injections can be performed either in a diagnostic way with the sole injection of a local anesthetic agent (e.g., to verify the correct level ahead of a planned surgery) or in a diagnostic and therapeutic way with an additional injection of steroids [27]. In our institution, the referring physician decides which structure should be targeted by the interventional radiologist based on the clinical symptoms of the patient and the imaging findings [28].

Safety is of course a crucial consideration for any medical procedure: CT-guided spinal injections are safe and reliable, with only rare adverse effects [29, 30]. When compared with fluoroscopic procedures, CT has some advantages such as better visualization of inadvertent intrafacet injection [19]. Specific risks are associated with the injection of corticosteroids in both CT-guided and fluoroscopy-guided procedures and are discussed in the last part of this review.

When choosing the image modality for spinal injections, radiation dose is an important factor. A decade ago, the effective radiation dose of CT (3.35 mSv) was substantially higher than that of fluoroscopy (0.85 mSv), especially when a full diagnostic lumbar CT scan was performed as part of the spine injection procedure [31]. However, even at that time, the effective radiation dose of the CT procedure itself was almost half of the fluoroscopic radiation dose (0.45 mSv vs. 0.85 mSv, respectively) because of the shorter fluoroscopy time at CT [31]. Based on these findings, it is advised to restrict the planning CT to the one or two spinal segments where the injection will be performed.

In our institution, a helical CT with a low-dose protocol limited to one or two spinal segments is performed for planning the procedure and to choose the optimal slice for the needle positioning. For the intervention itself, intermittent CT fluoroscopy with very low dose is used, leading to a slightly reduced image quality with more noise, but still adequate for needle placement and visualization of the injected contrast fluid.

A few years ago, we performed a comparison of the effective radiation dose for both the patient and the treating physician for fluoroscopy-guided vs. CT-guided spine injections: We found that while for the patients the effective radiation dose in fluoroscopy-guided injections was lower, for the treating physicians, the radiation dose with fluoroscopy was substantially higher than with CT [30]. For example, in fluoroscopy-guided lumbar transforaminal epidural injections, the radiation exposure of the patient was 0.24 ± 0.22 mSv; in the CT-guided intervention, it was 0.33 ± 0.10 mSv. For the interventionalist, the radiation exposure of the wrist in fluoroscopy-guided interventions was 1.44 × 10^−3^ ± 2.69 mSv and the radiation exposure of the body was 0.42 × 10^−3^ ± 0.99 mSv, while for the CT-guided interventions, the exposure of the wrist was 0.14 × 10^−3^ ± 0.55 mSv and that for the body was 0.11 × 10^−3^ ± 0.44 mSv [30]. Based on these data, we decided to use CT as the primary modality for the spine interventions, and to further optimize the CT protocols to reduce the radiation exposure of the patient.

**Fig. 1:**
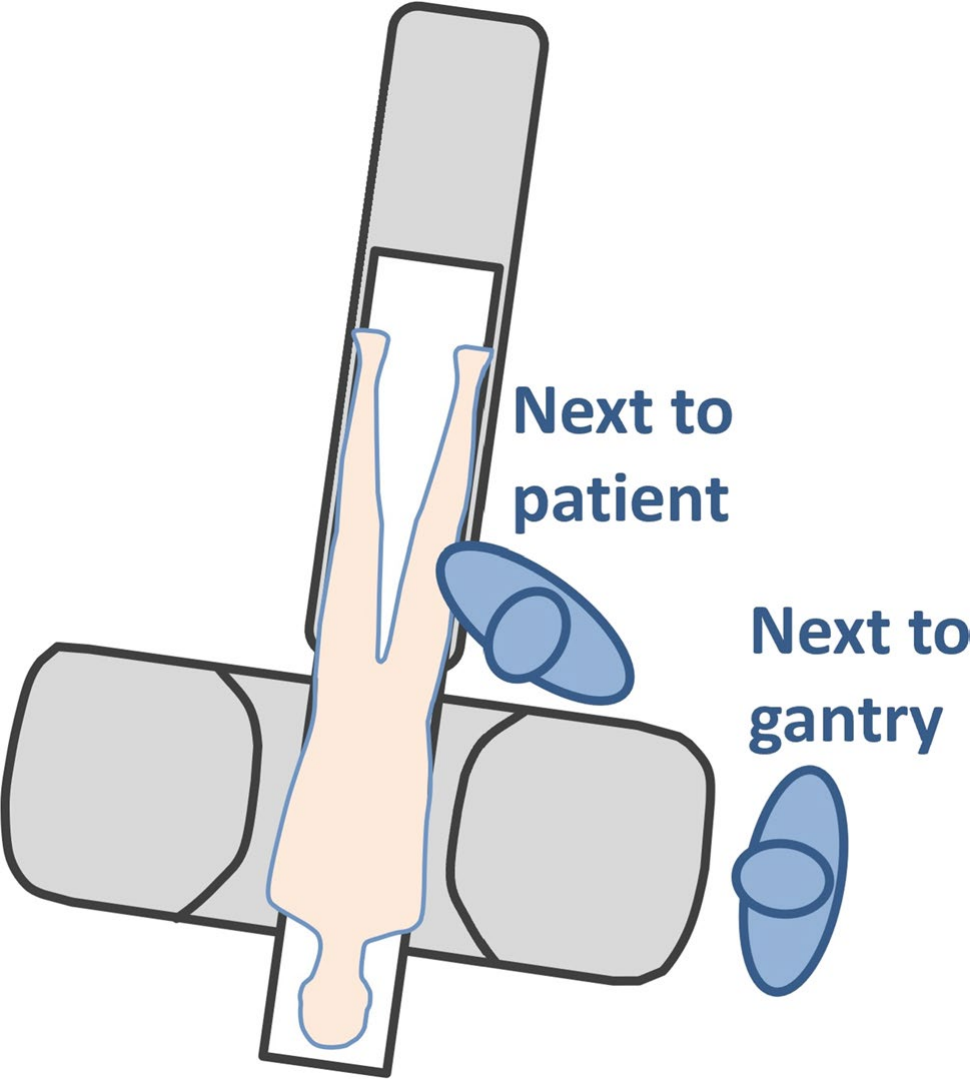
Illustration of the interventionalist’s position during CT-guided spinal injections. While previously many interventionalists were positioned directly adjacent to the patient during the whole procedure, it now has been recognized that next to the gantry of the CT the radiation exposure for the interventionalist is almost zero

Tin prefiltration with a tin filter placed between the x-ray tube and the patient for a spectral shaping of the x-ray beam is a promising method for reducing the radiation dose for both the patient and the physician during CT-guided injections. It allows reducing the radiation dose of a standard CT down to that of a radiographic examination, making it very beneficial for CT-guided spine injections [32]. The radiation exposure for the interventionalist can be reduced to zero if he takes a step back and stands behind the gantry [30] (Fig. 1). At our institution, we use both strategies, employing tin prefiltration for the CT-guided spine injections, and the interventionalist stands behind the gantry during intermittent CT fluoroscopy, whenever possible.

An alternative approach to substantially reduce the radiation dose is to omit the planning CT scan: A recent study in *Skeletal Radiology* evaluated the radiation dose for fluoroscopy-guided procedures versus ultralow-dose CT fluoroscopy, i.e., without a helical planning CT but using the scout for the planning of the injection level. Wieschhoff et al. found a much lower average dose for ultralow-dose CT fluoroscopy (0.15 mSv ± 0.11) versus fluoroscopy (0.30 mSv ± 0.34) [33]. This approach may work well for the majority of the patients, with the exception of patients that have substantial ossifications at the posterior spinal elements and/or postoperative patients, making a helical planning CT necessary prior to the CT-fluoroscopy-based lumbar spine injection. For safety reasons, we also recommend a short planning CT for all thoracic and cervical injections.

In the postoperative spine, the epidural fat is often missing, and the dura is directly adjacent to peridural scar tissue [34]. In such cases, CT is very helpful in depicting the precise anatomical situation, allowing the choice of a different entry location for epidural injections, rather than injecting into the scar tissue with the inherent risk of puncturing the dura.

In total, the considerations which modalities to use for spine infiltrations have changed during the last two decades: In the early 2000s, CT-guided procedures had a much higher radiation dose than fluoroscopy and often fluoroscopy was the preferred method. A number of technical innovations in the last two decades now allow CT-guided injections at a very low dose, thereby substantially reducing the radiation dose for the patient. And for the interventionalist, stepping back and positioning themselves behind the CT gantry result in an effective radiation exposure of zero, which is a clear advantage of CT over fluoroscopy, where the interventionalist is usually much closer to the patient.

At our institution, we perform spinal injections under CT guidance, as the intervention is simple and fast, allows a good visualization of the needle tip as well as of the location of the injected contrast, and is associated with a low radiation exposure for the patient and little to no radiation exposure for the interventionalist.

## CT-guided spinal interventions: how we do it

**Fig. 2:**
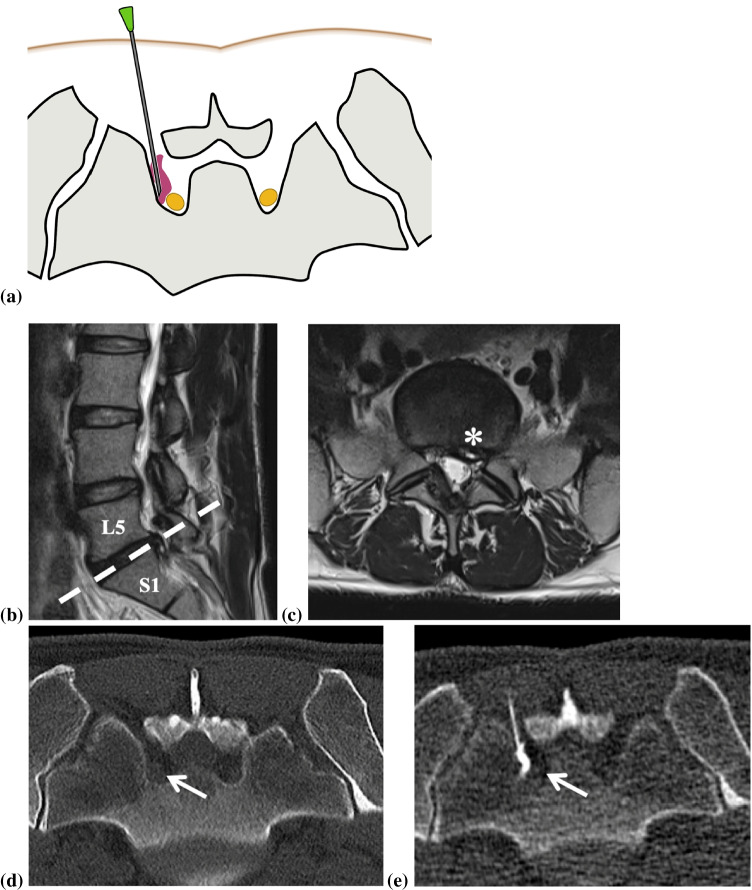
Illustration of a left-sided periradicular S1 nerve root infiltration (**a**) showing the position of the needle tip directly next to the left S1 nerve root (yellow) and adjacent contrast media dispersal (purple). A 36-year-old male patient, who had already undergone decompressive surgery in level L5/S1, presents with a hernia relapse (asterisk) and recurrent S1 radiculopathy on the left side (**b** sagittal and **c** axial T2-weighted image). CT-guided left-sided periradicular S1 nerve root infiltration was performed: The helical CT was used for planning (**d**), the navigation CT-fluoroscopy with a reduced radiation dose depicting the needle and the injected contrast touching the left S1 nerve (white arrow) (**e**). Note that for the MR examinations, the patient was in the supine position; for the CT-guided infiltrations, the patient was in the prone position

**Fig. 3:**
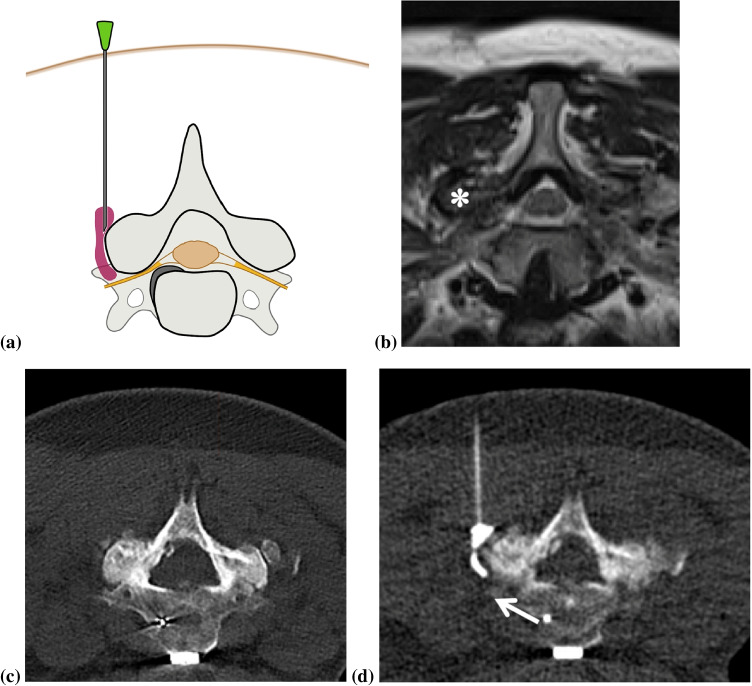
Illustration of a left-sided indirect C8 nerve root infiltration (**a**) showing the position of the needle tip posterior to the left facet joint C7/8 and contrast media dispersal (purple) around the facet joint reaching the left C8 nerve root (yellow). A 57-year-old female patient complaining about intermittent pain along her left dorsal arm and additional tingling paresthesia. An MRI of the cervical spine showed a severe osteoarthritis (asterisk) at the level C7/Th1 leading to high-grade foraminal stenosis (**b** axial T2-weighted image). After the helical planning CT (**c**), a CT-guided left-sided indirect C8 cervical nerve root block was performed. Note that for indirect cervical nerve root blocks, the needle is inserted from the posterior to the lateral aspect of the facet joint, and the contrast medium is dispersed around the facet joint, adjacent to the C8 nerve (arrow) (**d**)

**Fig. 4:**
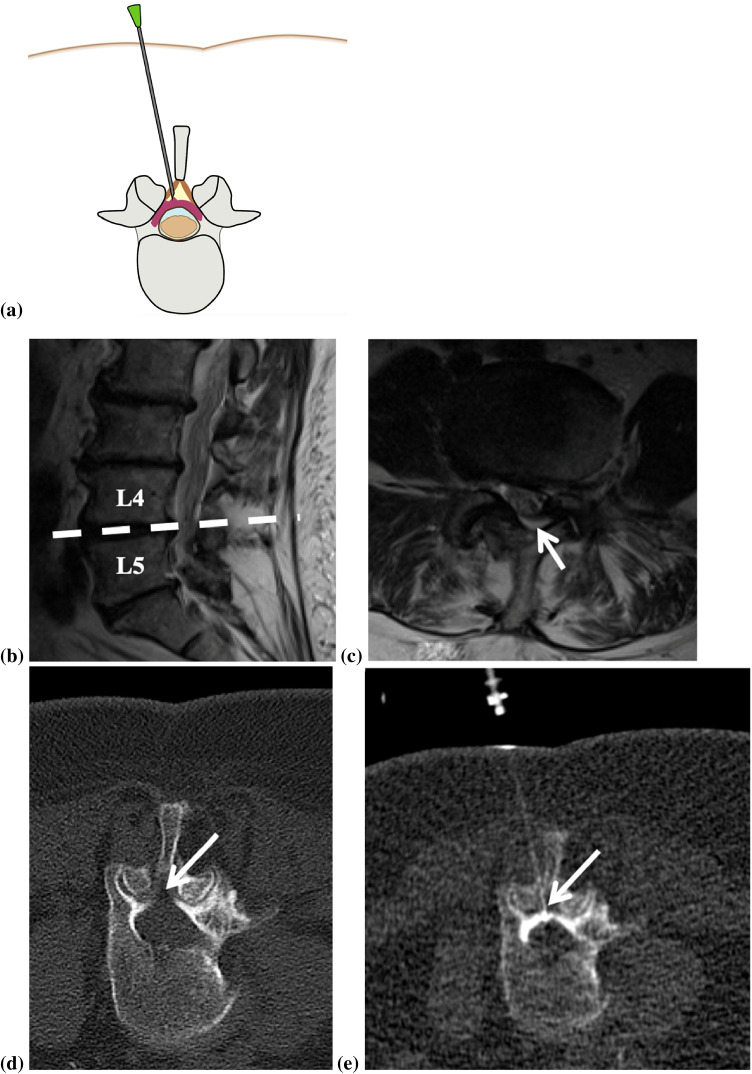
Illustration of an epidural steroid injection of the lumbar spine (**a**) showing the position of the needle tip in the epidural fat triangle (yellow) with contrast media dispersal (purple) in the epidural space along the dural sac (dark brown). A 70-year-old female patient with recurring lumbar back pain radiating down both legs. A sagittal T2-weighted MRI sequence of the lumbar spine showed multisegmental degenerative changes of the spine (**b**) that lead to a distinct narrowing of the spinal canal at the level L4/5 depicted on an axial T2-weighted image (**c**). Helical planning CT shows the epidural fat (arrow) which is the target zone for the needle tip (**d**). The patient underwent a CT-guided lumbar epidural infiltration at the level L4/5 with the needle tip in the epidural fat tissue (arrow) (**e**)

In our institution, all CT-guided interventions are performed in a highly standardized fashion with a 128-slice CT scanner (Somatom Definition AS; Siemens Healthineers, Erlangen, Germany), for nerve-root blocks and epidural injections (Figs. 2, 3 and 4).

In case of the use of anticoagulant medication, the intake of these drugs must be stopped before and after the intervention according to our internal guidelines. In our institution, the spinal injections are done without sedation, only with local anesthetics, as described below.

**Fig. 5:**
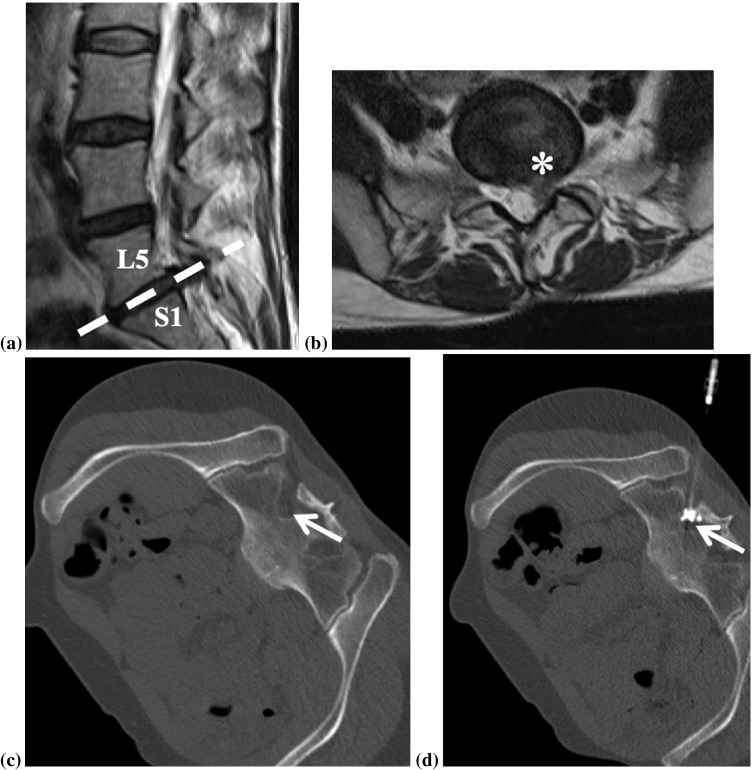
A 44-year-old female patient with a large disk herniation (asterisk) depicted on MRI at the level L5/S1, resulting in compression of the left S1 nerve root (**a**, **b**). Since the patient had severe pain in the prone position, she was slightly turned to her right side for the CT procedure, so she could better tolerate the periradicular S1 nerve root infiltration (**c**, **d**). The S1 nerve root in the CT images is marked with an arrow

**Fig. 6:**
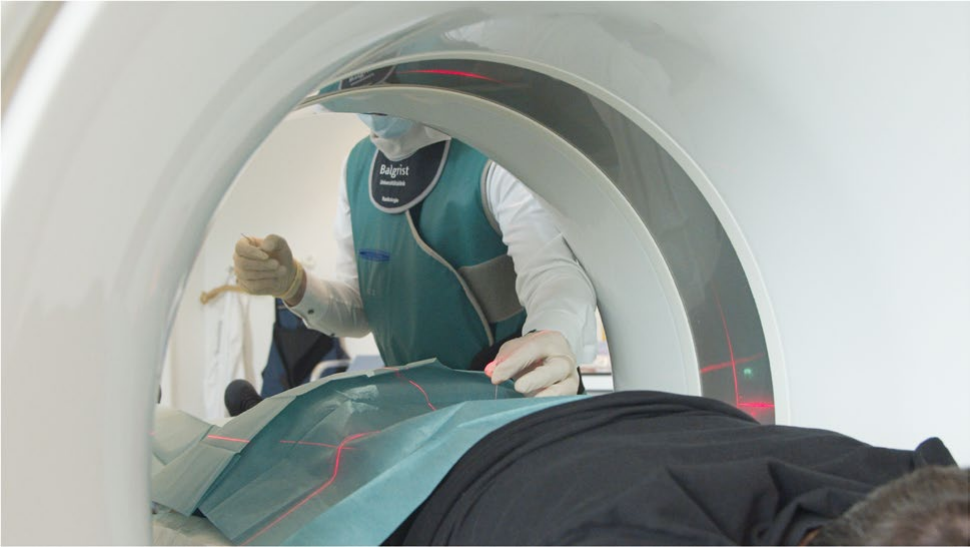
Radiologist performing a CT-guided corticosteroid injection in the lumbar spine. Patient placed in prone position with sterile sheet placed on the back. Laser navigation is used for precise needle placement

**Fig. 7:**
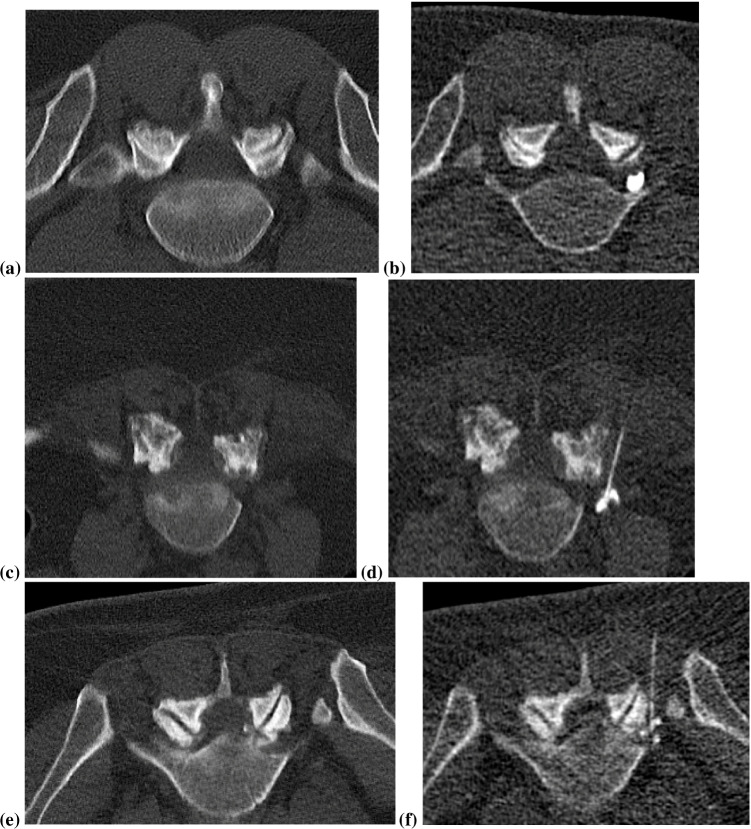
Examples of different contrast medium dispersal patterns in three different patients with right-sided periradicular L5 nerve root infiltration. On the left side of each row, the planning helical CT, on the right side the navigation CT-fluoroscopy image for needle placement, with more image noise due to the use of a low radiation dose CT protocol. **a**, **b** and **c**, **d** Focal non-linear contrast dispersal pattern. **e**, **f** Tram track contrast medium dispersal pattern

We perform spinal injections under CT guidance for the lumbar spine, the thoracic spine, and the cervical spine, and all with the patient in prone position. When performing a lumbar nerve root block or a lumbar epidural injection, a lateral CT scout view is obtained in the prone position with both arms next to the patient’s body. In rare cases where patients cannot tolerate the prone position because of severe pain, they can be slightly turned to either side for the duration of the CT procedure (Fig. 5). After acquisition of the short planning helical CT scan, the radiologist selects the adequate location for the interventional approach on a reconstructed axial plane and selects a needle with an appropriate length. For cervical and thoracic injections, needles with a caliber of 21G–22G are used, with a needle length of 4 to 8 cm. For lumbar injections, needles with a caliber of 20–21G are used, with a needle length of 7 to 12 cm. The skin is disinfected three times from inside to outside with Braunoderm (isopropanol and povidone iodine; B.Braun, Melsungen, Germany). A sterile cover with a central opening is then placed on the patient’s back, followed by the insertion and guidance of the needle in several steps (Fig. 6) under CT-fluoroscopy navigation. When the needle tip has been placed adjacent to the nerve root or in the dorsal epidural fat tissue, 0.5 ml of iodine contrast media (Iopamiro; iopamidol 200 mg/mL; Bracco, Milano, Italy) is injected to confirm the needle tip position next to the root/epidural, and to exclude an intravascular or intradural position. Then, the corticosteroid (1 ml of Triamcort (triamcinolone acetonide 40 mg/ml; Helvepharm, Frauenfeld, Switzerland) or Fortecortin (dexamethasone 4 mg/ml; Merck, Darmstadt, Germany)) and 1 ml of 0.2% ropivacaine (ropivacaine 2 mg/ml; Sintetica, Mendrisio, Switzerland) is injected. Interestingly, the therapeutic outcome is independent of the dispersion pattern along the nerve root or the location of the contrast agent, as recently shown by Germann et al. [26] (Fig. 7).

When performing interventions of the cervical or thoracic spine, important and special issues have to be taken into consideration: For cervical nerve root blocks (and the very rare thoracic nerve root blocks), for safety reasons, we use solely an indirect approach for the infiltration, inserting the needle from dorsal with the needle tip on the lateral aspect of the facet joint. When the needle tip is not placed adjacent to the cervical nerve root, these indirect cervical nerve root blocks yield a good short- and long-term pain relief [35].

**Fig. 8:**
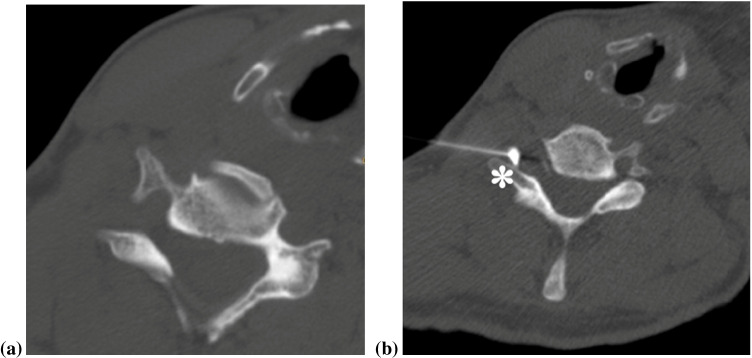
Example of the direct nerve root injection technique for the cervical spine which was abandoned in our institution more than a decade ago due to safety reasons. CT-guided left-sided direct C6 cervical nerve root block in a 43-year-old female patient. The patient is in a supine position, with the head slightly tilted to the contralateral side. After a planning CT (**a**), the needle was inserted from the lateral side, and the position of the needle tip next to the cervical nerve root (asterisk) was verified with a contrast injection (**b**)

Why do we perform an indirect approach rather than a direct intraforaminal placement of the needle for cervical nerve root blocks? Complications of these direct intraforaminal cervical injections have been reported especially when using particulate steroids [11, 36]: Brain and spinal cord infarctions were triggered by cervical injections of particulate steroids, likely because of clotting of steroid particles when inadvertently tapping periradicular arteries. Because of these risks, we no longer perform the direct approach in cervical and thoracic infiltrations (Fig. 8). Furthermore, for lumbar injections with levels above L4, we do not use particulate steroids, as described in detail in the next section*.*

### Pain assessment

To evaluate the effectiveness of the injection procedure, our patients are asked before and 15 min after the intervention to assess their pain level on the Numeric Rating Scale from 0 to 10 [37]. This information is added to the radiological report about the intervention and is a helpful diagnostic information for the referring orthopedic surgeon or rheumatologist. Further, in the rare case of a serious adverse event, it is helpful that the patient stays in our department for 15 min after the injection and may be transferred to the emergency department without any delay, if necessary.

## Risks and side effects

### Corticosteroid-related risks

After the U.S. Food and Drug Administration (FDA) reported neurologic adverse events following epidural administration of corticosteroids, in 2014, it published a safety announcement and warned of adverse events, including loss of vision, stroke, paralysis, and death [38]. In May 2015, the FDA defined safeguards in collaboration with a working group to favor the use of non-particulate corticosteroids and to navigate needle placement with imaging guidance [39]. However, in a later publication, the FDA clarified that non-particulate corticosteroids are not safer than particulate corticosteroids [40]. Manchikanti et al. criticized the warning of epidural injection of corticosteroids by the FDA for not being evidence-based [41]. There are multiple studies showing that the use of particulate instead of non-particulate corticosteroids for spinal injections is more effective: The patients reported short-term as well as long-term pain relief and a higher pain reduction [42–44]. In a study with 531 patients, Bensler et al. showed that particulate corticosteroids led to a significantly higher pain reduction, and that patients treated with non-particulate corticosteroids reported a significant worsening of their symptoms [43].

At our institution, we use two types of corticosteroids for spinal injections: Triamcort (triamcinolone acetonide 40 mg/ml; Helvepharm, Frauenfeld, Switzerland) or Fortecortin (dexamethasone 4 mg/ml; Merck, Darmstadt, Germany). For nerve root blocks and epidural injections below the level of L4 and lumbar facet joint injections, we offer our patients to inject the particulate steroid Triamcort in an off-label use based on the significantly higher pain reduction compared to non-particulate steroids, and if the patients agree, they sign a consent form. For the spinal injections above the level of L4, we use the non-particulate corticosteroid Fortecortin.

For repeated epidural steroid injections, a cumulative triamcinolone dose of 400 mg was shown to reduce bone density in postmenopausal women [45]. Therefore, it has been advised to limit the corticosteroid injections to one injection every 6 weeks or to three to four injections during 1 year [46].

Another known side effect of corticosteroid injections is a flare reaction whereby shortly after the infiltration of corticosteroids the patient experiences worsening of pain. However, this effect subsides after 2 to 3 days and it has not been shown to influence the therapy outcome [47].

### Hematoma

Spinal epidural hematoma is a rare complication after spinal injections; there are reported cases where patients had to undergo surgical decompression [48]. In a case report, Benzon et al. recommend caution when performing epidural injections in patients receiving antiplatelet drugs [49]. For our institution, guidelines for stopping medication pre- and postinterventionally were developed for patients with blood-thinning medications or bleeding tendencies. If a patient is taking acetylsalicylic acid (e.g., aspirin) at a dose of 100 mg a day or less, the spinal injection will be performed without stopping the medication. If the dose of acetylsalicylic acid is higher or if another antiplatelet or anticoagulant medication is taken, the medication will generally be stopped or modified. In our institution, there is no known case of a clinically relevant hematoma after spinal injections.

### Infection

When performing a standardized, careful, and aseptic procedure, we experienced an extremely low risk of infection. The risk of postoperative surgical site infection is significantly increased to an infection rate of 2.2% if lumbar epidural corticosteroid injections are performed 1 to 3 months prior to surgery [50]. If the time interval between epidural injection and surgery was longer than 3 months, the infection rate dropped to 1.5% and there was no difference compared to the control group which did not receive an epidural corticosteroid administration [50].

## Conclusions

CT-guided spinal injection of corticosteroids is a safe and effective minimally invasive treatment method with a low radiation dose exposure. The injections can lead to a significant relief in back pain, and successful injections may delay the need for decompressive surgery. The use of CT for spinal injections allows a direct visualization of the nerve root and the needle tip, as well as of the distribution pattern of the injected contrast.
